# Reconfigurable artificial neuron and synapse enabled through a single alloyed memristor

**DOI:** 10.1038/s41598-025-15251-x

**Published:** 2025-08-13

**Authors:** Elias Passerini, Mila Lewerenz, Arnaud Schneuwly, Nadia Jimenez Olalla, Markus Fischer, Raphael Gisler, Luiz Felipe Aguinsky, Alexandros Emboras, Yuriy Fedoryshyn, Mathieu Luisier, Thomas Schimmel, Miklós Csontos, Ueli Koch, Juerg Leuthold

**Affiliations:** 1 Institute of Electromagnetic Fields (IEF), ETH Zurich, Zurich, 8092 Switzerland; 2 Integrated Systems Laboratory (IIS), ETH Zurich, Zurich, 8092 Switzerland; 3 Institute of Applied Physics (APH), Karlsruhe Institute of Technology, 76131 Karlsruhe, Germany; 4 DeepNano Group, James Watt School of Engineering, University of Glasgow, Glasgow, G12 8LT UK

**Keywords:** Electrical and electronic engineering, Electronic and spintronic devices, Computer science, Electrochemistry

## Abstract

Memristive devices have drawn significant interest due to their use in novel paradigms such as neuromorphic computing. Neuromorphic systems are developed by implementing artificial neurons and synapses on a hardware level. Hence, memristors with multipurpose and reconfigurable neuromorphic functionalities could be highly beneficial in the design process. In this study, we experimentally verify that both neuronal and synaptic functions can be implemented on a single memristor. By controlling the device current at two different levels, the memristor operates in either a volatile or a nonvolatile retention regime. These two operation regimes are essential to mimic neuronal or synaptic behavior. Towards this end, we use an alloyed filamentary memristor (AgSn/SiO_2_/Pt) composed of ions with differing mobilities enabling both integrate and fire (IF) operation in the volatile regime and synaptic weights in the nonvolatile regime. By only changing the current compliance, these devices switch reliably between the aforementioned retention regimes. Additionally, our proposed training method significantly improves switching variability in the volatile regime. We show how the mean set voltage statistically reduce from 1.2 to 0.2 V; and the standard deviation of the set voltages reduced from 0.52 to 0.03 V.

**Supplementary Information:**

The online version contains supplementary material available at 10.1038/s41598-025-15251-x.

## Introduction

The demand for fast and energy efficient computation is steadily increasing due to the development of technologies such as machine learning and artificial intelligence^1^. Classical approaches, which rely on the von Neumann architecture, where memory and processing are separated, might not be enough to meet this demand in an ecological and sustainable way^2^. Therefore, new paradigms are researched to solve the shortcomings of classical architectures. Neuromorphic computing is a new discipline investigating such new paradigms^3^. Being inspired by the human brain, neuromorphic architectures promise similar energy efficiency as their biological counterpart. These architectures could profit from components, which are able to implement directly the desired biological functions in hardware. Ideally, these components are multipurpose and reconfigurable. Memristors represent promising candidates to implement the neuromorphic circuits. They have already been shown to implement some of the desired biological functions^4,5^.

So for instance, memristive devices have found wide application in neuromorphic computing^3,6–11^. While volatile memristive devices lend themselves to the implementation of artificial neurons, nonvolatile ones are excellent candidates for weights in electrical synapses^7^. Various neuron functions have already been demonstrated with different memristor materials. For example, NbO_2_ memristors have been reported to exhibit 15 types of neuron like dynamics^12^. Even 23 neuron behaviors have been recently shown with two VO_2_ memristors emulating most of the known biological neuronal dynamics^4^. Other types of memristors have been used to demonstrate synaptic emulations^8,13–15^. Synaptic properties such as short-term and long-term plasticity have been shown with Ag_2_S^16^. Additionally, nonvolatile memristors are also able to emulate second-order synaptic functions^17^. Multipurpose functions such as the coexistence of threshold and memory switching have been shown with HfO_2_ devices^18^. Only recently, reconfigurable multipurpose memristor have found wider interest. Halide perovskite nanocrystals are able to switch between volatile and nonvolatile modes^19^. For Ag/SiO_2_ memristors, two types of integrative effects have been shown in the HRS and LRS which could be a useful feature for synaptic and neuron circuits^20^. Ag/SiC/n-Si memristors have shown to implement long and short term memory and with additional circuit elements also leaky integrate and fire^21^. Synaptic and nociceptive behavior using volatile and nonvolatile switching has been demonstrated with a TiO_2_ memristor^22^. Implementation of both neuron functionalities and synaptic weight under bias has been shown with a reconfigurable Ag/MoS_2_/HfAlOx/cnt textile network^23^. Artificial synapses and neurons have been demonstrated by using Ag-nanoclusters devcies^24^. Beyond memristors, other technologies have proven to be able to implement neuron and synaptic functions such as MoS_2_ Neuristors^25^. However, despite of the availability of materials, systems which can emulate both neuron and synapse in simple and controlled manner with a single memristor, are still sought after. Ideally, one should be able to switch between neuron or synapse operation and vice versa without an extensive control circuitry. Thereby, meeting the increasing demand for multipurpose and reconfigurable analog platforms^26^.

In this work, we experimentally verify that a single AgSn-alloyed filamentary memristor in series with a resistance is able to emulate both neuron and synapse-like operations. These devices have been explored in a previous work, which investigated the effect of alloyed electrode materials and compliance current on the reliability of the device^27^. By controlling the compliance current, we can choose the operation mode. We demonstrate neuron-like functionalities such as All-or-None, Integrate, and Integrate and Fire (IF) with refractory period on a single device. Additionally, we provide evidence that the same device can be used for synaptic weights. To mimic both neuron and synaptic functionalities, the device is operated in two different regimes. The first regime is a volatile regime at a low compliance current (in the order of nA). The short-term stability of the filament in this operation regime makes it a viable approach to realize neuron-like functionality. So for instance, this can be exploited to implement the IF operation of a neuron. The second regime is a nonvolatile regime at a higher compliance current (mA). Applying higher currents leads to the formation of a long-term stable filament. This long-term stability is ideal for the realization of different conductance levels, which can be used as synaptic weights. In addition, we show that our devices can change back and forth between these two operations regimes. Another finding relates to the reduction of the set voltage and impressive improvement of the cycle-to-cycle variance of the volatile device performance. It is found that after an initial training of the device in its non-volatile state, the mean set voltage of the volatile state reduces in the best case from 0.77 V to 0.23 V and its standard deviation improves from 0.22 V to 0.01 V.

## Concept

To demonstrate the implementation of both an artificial neuron and synapse we use a single AgSn-alloyed memristor. This device enables neuron as well as synapse-like operation modes with a single memristive device by only varying the input signal in combination with a series resistance. Towards this end, it is important to identify devices that offers both reliable volatile and non-volatile operation modes.

One way to access these different operation modes is by utilizing the filament relaxation time. This relaxation time can be influenced by various parameters as previously shown. Ag based memristors have an input voltage dependent relaxation characteristics^28^. During relaxation the Ag filament turns into a chain of nanoclusters^29^. Depending on the filament strength during formation one can control the relaxation time of the metallic filament. One way to influence the filament shape is by material choice^30^. It has been shown that alloying can be used to improve synaptic weights^31^ and stabilize non-volatile operation^27^. In the first case an Ag electrode was alloyed with Cu, in the 2nd case an Ag electrode was alloyed with Sn. Simulations indicate that Sn ions are less mobile than Ag ions, as seen in Supplementary Fig. 2. Therefore, Sn likely contributes to stability during non-volatile operation, while mobile Ag ions dominate the volatile behavior. Additionally, it is possible to control the strength of the filament with the pulse amplitude and the compliance current^18,27,32–35^. A thorough investigation of the influence of the series resistance on the relaxation time shows a strong dependence and suggests the possibility to tune the relaxation behavior using series resistances^36^. This ability to tune the relaxation time of Ag based memristors has been used to implement various synaptic behaviors such as short and long-term synaptic plasticity^16,32,35,37^ and spike-timing dependent plasticity^18^. The electrochemical and thermodynamic processes of metal nanoclusters have been shown to enable both synaptic and neural operation with an Ag based memristor^24^. In this work, current limitation is used as a tuning mechanism to choose the desired neuron and synapse-like operation on the same device. Retention measurement for the two operation modes can be found in Supplementary Fig. 3.

To find parameters for the different operation modes, an initial characterization of the devices for the pulsed regime has been performed. The relation between the delay and the set pulse amplitude was investigated. The measurement revealed an exponential dependency between these parameters, see Supplementary Fig. 4. This relation has been shown for other Ag based memristors^38,39^. An increase of the input pulse voltage leads to a shortening of the delay time when the device turns from the HRS to the LRS. Additionally, the relaxation time of the filament when it changes from the LRS to the HRS can be influenced by the pulse length^39^.

**Fig. 1:**
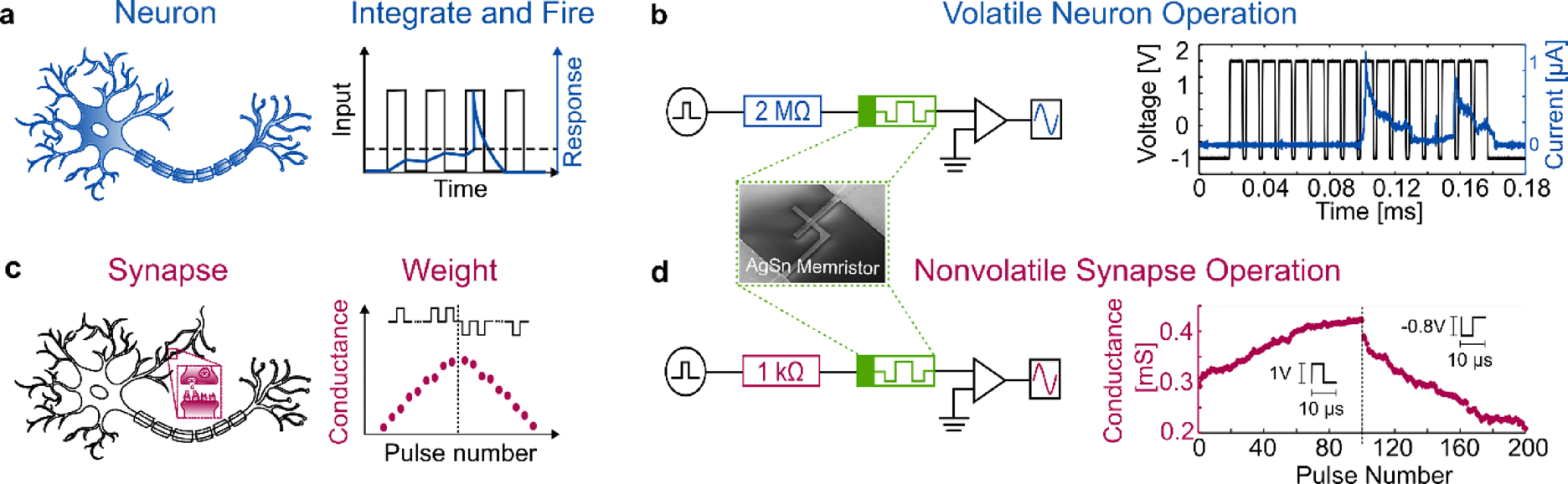
**a** single alloyed memristor emulating both neuron and synaptic functionalities. (**a**) A neuron and the Integrate and Fire (IF) operation for neurons. After integrating the input signal (black), a spike response (blue) is triggered. (**b**) Implementation of an IF operation mimicked by a simple circuit consisting of a series resistance (2 MΩ) and an alloyed AgSn memristor. The high series resistance limits the current and therefore allows for operation in the volatile regime of the device. A rectangular voltage pulse train (black) with a bias offset is applied. The current (blue) through the device shows a spiking and subsequent refractory period at the 9th and 14th pulses of the applied voltage similar to the IF operation. (**c**) A synapse and visualization of synaptic weight function. A train of positive pulses (black) increases the conductivity (red) while the subsequent train of negative pulses decreases it. (**d**) Implementation of synaptic weight with a simple circuit consisting of a low series resistance (1 kΩ) and the same alloyed memristor. Due to the low series resistance and therefore high currents, the device is operated in a nonvolatile regime. The conductance of the device (red) shows an increase with positive pulses and a subsequent decrease with negative pulses similar to synaptic weights.

To show neuron-like functionalities, we implemented an Integrate and Fire (IF) operation. A schematic of a neuron together with this firing mechanism is depicted in Fig. 1a. In this operation mode, the input signal consists of a pulse train (black) which triggers a neuronal spike response (blue). The spiking event is followed by a refractory period, which emulates the diffusion of ions through the cell membrane, after which the neuron returns to the initial state. We show the implementation of an IF operation in a simple circuit consisting of a 2 MΩ resistance in series with an alloyed (AgSn) memristor, Fig. 1b. A transimpedance amplifier is used to measure the current through the memristor. More details on the measurement setup can be found in the electrical measurement and data analysis section. Due to the inherent current limitation of the series resistance, the memristor is operated in the volatile regime. A voltage pulse train (black) with an amplitude of 3 V operated around an offset of − 1 V is used to trigger the spike response of the memristor. As can be seen by the current through the memristor (blue), the spiking event is triggered at the 9th and 14th pulse of the pulse train. This response is the result of the memristor shortly changing from a high resistance state (HRS) to a low resistance state (LRS), thus allowing more current passing through the device. Each spike is followed by a refractory period where the current through the memristor reduces, indicating a return of the memristor to a HRS. Therefore, neuron-like functions can be implemented with a simple circuit consisting of a series resistance and an alloyed memristor.

An important property of synapses is the synaptic weight. It refers to the strength of the connection between two nodes. This synaptic weight is adaptable and can be increased (potentiation) or decreased (depression). A synapse and its synaptic weight are illustrated in Fig. 1c. A positive pulse train (black) increases the conductance/weight of the synapse (red dots) while a negative pulse train decreases it again. The experimental implementation of synaptic weights is demonstrated in Fig. 1d. The setup consists of the same components as for the neuron measurement with the only difference being the lower series resistance (1 kΩ), which allows operation of the memristor in a nonvolatile regime. This is necessary for the implementation of long-term synaptic weights. As an input signal (black) a voltage pulse train of 100 potentiations (1 V, 10 µs) followed by 100 depressions (− 0.8 V, 10 µs) is chosen. The conductance (red dots) of the memristor increases during potentiation pulses and decreases during the depression pulses, thus implementing synaptic weights with a simple memristive circuit. In conclusion, we verify the possibility to implement both the IF operation and synaptic weights on a single device in a simple experimental circuit by operating under volatile or nonvolatile conditions, respectively.

## Mimicking neuron operation

In this section, we discuss the implementation of three different neuron functions: “All-or-none”, “Integrate”, and “Integrate and Fire”. These functions are demonstrated with a simple circuit consisting of a series resistance and a memristor. The role of the series resistance is to limit the current through the device, thereby ensuring volatile operation.

**Fig. 2:**
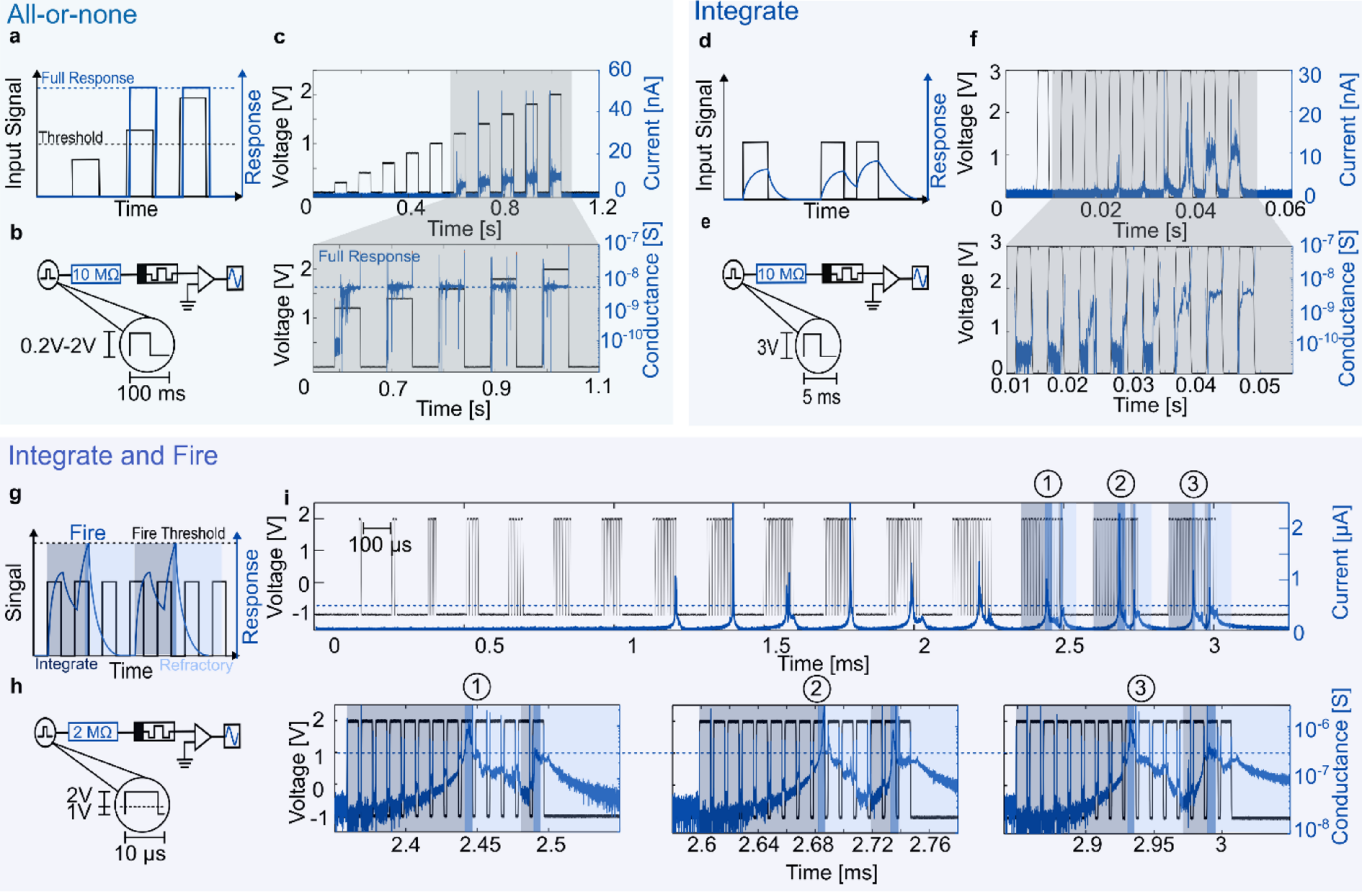
Neuron functions, “all-or-none”, “integrate”, and “integrate and fire”, implemented with a series resistance and a memristor. (**a**) “All-or-none” describes the “full response” of a neuron when the input signal is above a threshold. (**b**) Setup and input pulse shape for the “all-or-none” experimental measurement. (**c**) The input voltage consists of 10 pulses with increasing amplitude (black). The memristor turns on at a threshold of 1.2 V which can be seen by the sudden increase in current (blue) at the 6th pulse. The zoom-in shows the conductance of the device for each of the pulses above the threshold. One can see a “full response” in the conductance for all pulses. (**d**) The “integrate” function (blue) of a neuron is visualized for different timed input pulses. (**e**) Setup to measure the integration capabilities of the memristors with shown pulse signals. The pulse duration was reduced to 5 ms compared to the “all-or-none” measurement. (**f**) A voltage input pulse train consisting of 9 pulses is shown in black. As can be seen by the increasing current through the device (blue) the memristor is able to integrate. This can also be observed in the zoom-in where the conductance of the device increases with increasing pulses. (**g**) ”Integrate and Fire” (IF) operation for a neuron visualized. After an integration section (dark blue shade), the neuron reaches a firing threshold resulting in a spike (blue shade). This spike is followed by a refractory period (light blue shade) where the neuron recovers. After the recovery, the neuron is ready to “integrate and fire” again. (**h**) Setup and input pulse shape for IF measurements of the alloyed memristor. (**i**) Pulse packages with intervals of 100 µs and an increasing number of pulses are sent from the source e.g. one pulse in the first pulse package, two in the second, etc. The current through the device spikes at the 8th/9th pulse of a package and again at the 14th pulse. The zoom-in shows the conductance of the memristor for the last three packages. The integration can be observed by the conductance increasing at the beginning of the package (dark blue shade). After integration and upon reaching a threshold (dashed line), the spiking event (blue shade) takes place. The device then recovers in the refractory period (light blue shade).

As a first neuron function “All-or-None”, is demonstrated, see Fig. 2a–c. Towards this operation, a pulse train with increasing amplitude is used as input signal. Upon reaching an input amplitude threshold, the neuron should react with a “full response”. Full response means that the response has the same magnitude for each input pulse above the threshold independent of the input amplitude, Fig. 2a. Such a threshold function is essential for the implementation of integrate and fire neuron models^40^. In the case of the experiment the full response can be seen in the conductance of the device reaching similar values for all voltage input amplitudes above the threshold. The experimental circuit consists of a resistance (10 MΩ) in series with the alloyed memristor, Fig. 2(**b**). Based on the previous amplitude measurement (Supplementary Fig. 4) a sweep from 0.2 V to 2 V was chosen with a pulse length of 100 ms. A transimpedance amplifier is used to measure the current through the device. The experimental results are shown in Fig. 2c. 10 voltage pulses with increasing amplitudes from 0.2 V to 2 V (black) are used as an input signal. As the switching voltage of this material stack is typically below 2 V. The pulse length is chosen to be 100 ms with a duty cycle of 50%. The threshold of the circuit is around 1.2 V as can be seen by the sudden increase of current (blue) at the 6th pulse of the pulse train. Subsequent pulses with higher amplitude also trigger the current response. A zoom-in to the switching pulses shows how the device reaches a “full response” conductance for each of the different input pulses above the threshold. A linear plot of the measurement can be found in the Supplementary Fig. 5.

Second, we test the device for its neuronal “integration” functionality, Fig. 2d–f. For this operation, the input signal (black) should lead to an increase in the memristor current (blue), Fig. 2d. The measurement circuit is shown in Fig. 2e. In our implementation, the input signal is a pulse train with a voltage amplitude of 3 V and a length of 5 ms (black) and a duty cycle of 50%, Fig. 2f. Compared to “All-or-None” measurement in Fig. 2a–c, the pulse length has decreased to highlight the integration. The current through the device increases nonlinearly over multiple pulses indicating an increase of the conduction state of the memristor (zoom-in). This measurement shows that a voltage pulse train can gradually decrease the resistance state of the memristor by selecting a proper cycle frequency. Therefore, it can be used to emulate a non-linear integration operation without a need for the typical parallel capacitance.

Finally, we test the alloyed memristor for its “integrate-and-fire” (IF) functionality, Fig. 2g–i. A schematic of the behavior is shown in Fig. 2g. In this operation mode the input signal consists of voltage pulse trains (black). The response has been visualized in blue. First, the voltage input signal is integrated (dark blue shade) and upon reaching a threshold, a spiking event takes place (blue shade). After the spiking, the device response mimics the refractory period of a neuron where arriving input pulses cannot trigger a spike for a while (light blue shade). The experimental setup is shown in Fig. 2h. The series resistance of 2 MΩ was again chosen such that volatile operation is ensured. The positive input voltage pulse amplitude has been set to 2 V which was followed by a negative − 1 V. The combination of negative bias (− 1 V) and increased duty cycle (80%) resulted in short negative biased sections between the input pulses. These sections were necessary to ensure significant filament relaxation between spiking events. This relaxation helps emulating the refractory period of a neuron where it is unable to fire despite receiving input pulses. Smaller bias offsets (− 0.5 V) were not enough to allow for a second spiking event in the given pulse scheme, see Supplementary Fig. 7. The experimental results are shown in Fig. 2i. The input signal (black) consists of voltage pulse packages with an increasing number of pulses. One pulse in the first package, two pulses in the second and so on. The interval between pulse packages is 100 µs. The first spiking event seen in the device current (blue) happens on the last pulse of the 8th pulse package. In subsequent pulse packages, a first spiking event takes place during either the 8th or 9th pulse followed by a second spike at the 14th pulse. The current spikes are the result of a sudden decrease of the memristor resistance as can be seen by the zoom-in to the last three pulse packages. In more detail, at the start of a pulse package the device integrates (dark blue shade) the input signal by increasing the device conduction. Upon reaching a threshold (dashed line), the memristor turns on, which can be seen by the spiked increase in conduction (blue shade). The spiking event is followed by a refractory period, in which the memristor returns to a higher resistance state (light blue shade). After the refractory period, the device is again ready to integrate and spike. This measurement shows that it is possible to implement an IF operation with only a series resistance and a memristors and without the need of an additional capacitance for the integration. Additionally, due to the tunability of memristors one can emulate spiking with different experimental parameters as shown in Supplementary Figs. 6, 7.

## Mimicking synaptic operation

In this section, the experimental verification of synaptic weights with the same alloyed memristor, as previously used for the neuronal-like functions, is discussed. Additionally, potentiation and depression over multiple cycles is investigated.

**Fig. 3:**
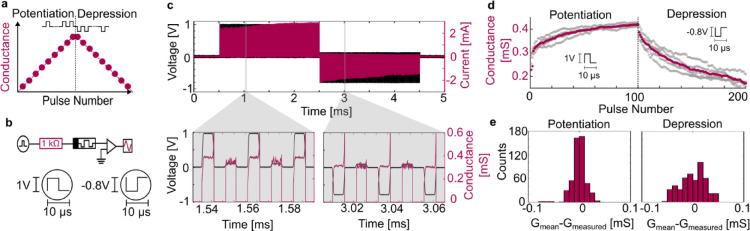
Synaptic operation of the alloyed memristor. (**a**) A synapse increases its weight/conductance (red) with each positive input pulse (Potentiation). A negative input pulse will decrease its weight/conductance (Depression). (**b**) Setup and input pulse for demonstrating the nonvolatile synaptic operation of the memristor. (**c**) 100 positive input pulses followed by 100 negative input pulses are sent from the source (black). After each pulse, a read signal with 0.1 V amplitude is applied (zoom-in). Following an initial fast turn on, the on-state conductance of the memristor is changed as can be seen by the current through the device (red). (**d**) Mean conductance levels during the read pulse of the input signal shown for five measurements. The calculated mean is shown in red. (**e**) The difference of the measured conductance $\:{G}_{measured}$ from the mean calculated conductance $\:{G}_{mean}$. The plot shows the difference of all five operation with 100 points each. One can see a high repeatability of the synaptic weight measurement for both potentiation and depression.

Synaptic weight updates are an essential part of synapses. An ideal representation of the synaptic weight functionality is shown in Fig. 3a. A train of positive pulses (Potentiation) gradually increases the conductance (i.e., synaptic weight). A subsequent series of negative pulses (Depression) decreases the conductance again. The experimental setup to realize synaptic weights is shown in Fig. 3b. It uses the same alloyed memristor as in the previous measurements. The current limitation is implemented by a low series resistance (1 kΩ), thus currents in the device are in the mA range. In this range, the memristor operates in the nonvolatile regime, which is desirable for emulation of long-term synaptic weights. Figure 3c shows the experimental synaptic weight measurement. The input signal consists of 100 positive pulses with an amplitude of 1 V followed by 100 negative pulses with an amplitude of − 0.8 V. The current through the device (red) shows a gradual increase for the positive pulses and a gradual decrease for the negative ones. A read pulse is used to evaluate the conductance after each signal pulse (see magnified view of Fig. 3c. Taking the mean conductance on each read pulse, five subsequent synaptic weight measurements are evaluated, Fig. 3(**d**). These measurements are shown in grey and the calculated mean conductance value $\:{G}_{mean}$ for each pulse is shown in red. The potentiation and depression curves are not symmetric. A possible reason could be the different processes happening during the increasing and decreasing of the filament conductance. One observes a low variability between cycles. The variation from the calculated mean conductance for potentiation and depression is small with a standard deviation of 15.97 µS and 10.86 µS respectivly, see Fig. 3e.To conclude, this experiment shows that besides neural functions, the alloyed memristors has also the ability to implement synaptic weights by adapting the series resistance.

## Changing operation mode

In this section, we demonstrate a training method to improve the cycle-to-cycle variability in the volatile regime. Additionally, we investigate the ability of the alloyed memristors to switch between the volatile and nonvolatile operation regimes.

**Fig. 4:**
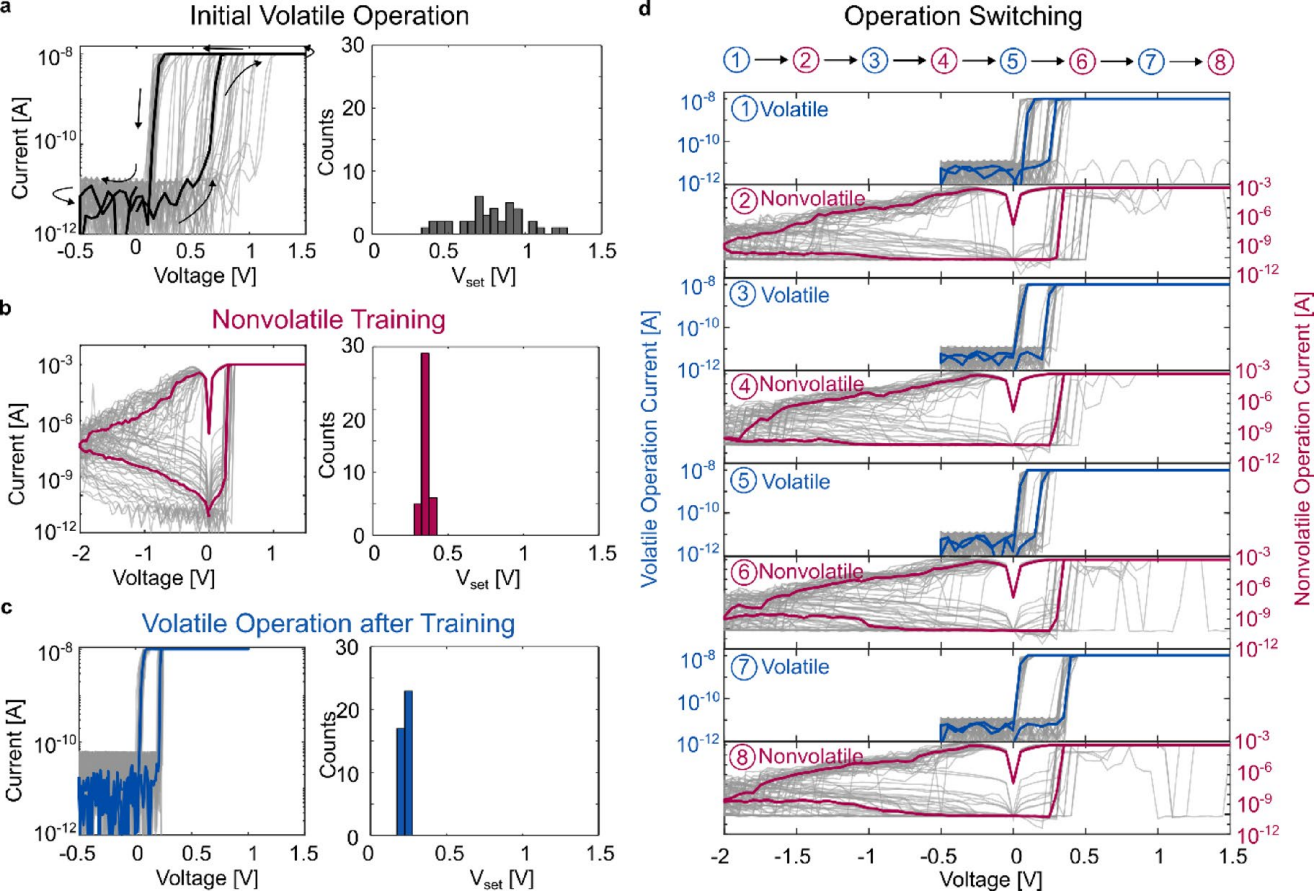
Training and switching between volatile and nonvolatile operation of the alloyed memristor. (**a**) Volatile I(V) cycles as found during the initial training cycles. The plot shows 40 $\:I\left(V\right)$ cycles in light grey with a current limitation of 10 nA and the corresponding Vset distribution. The sweep direction of the cycles is indicated by the arrows. The median $\:I\left(V\right)$ cycle is highlighted in dark. The Vset distribution during the initial volatile operation is broad (**b**) Nonvolatile training with a current compliance of 1 mA. 40 $\:I\left(V\right)$ cycles are plotted in grey with the median calculated cycle highlighted in red. In the nonvolatile operation regime, the device exhibits a narrow set voltage distribution. (**c**) Highly repeatable volatile cycles as found after training in the nonvolatile regime. 40 $\:I\left(V\right)$ cycles are plotted with the median highlighted in blue. After cycling in the nonvolatile operation regime, the volatile operation drastically improves compared to the initial volatile cycles. (**d**) Switching seven times back and forth between operation regimes with an alloyed memristor. Each subfigure consists of 40 cycles (light grey) with the calculated median cycle highlighted (blue or red). This proves the ability to change the operation regime by only adapting the current limitation.

To show the improvements gained by the proposed training method, cyclic voltammetry ($\:I\left(V\right)$) measurements are used. A source measurement unit (SMU) is directly connected to the memristor and applies a triangular voltage signal. The voltage is swept from 0 V to 1.5 V, to − 0.5 V and back to 0 V with a sweep speed of 1 V/s. In the initial volatile operation, the current is limited to 10 nA. This initial volatile operation is shown in Fig. 4a. 40 $\:I\left(V\right)$ cycles (grey) and the calculated median cycle (black) are plotted from − 0.5 V to 1.5 V. The arrows indicate the sweep direction. The volatile character of the device can be seen by the low current in the second part of the measurements while sweeping the negative polarity. The set voltage (V_set_) distribution, where the memristor turns from a high resistance state (HRS) to a low resistance state (LRS), is shown in the histogram. The distribution has a wide spread from 0.35 V to 1.25 V with a mean of 0.77 V and a standard deviation of 0.22 V. The evolution of mean set voltage and the standard deviation over these 40 cycles can be found in Supplementary Table 1. After this initial volatile operation of the memristor, the current limitation is steadily increased, and the device is repeatedly cycled until it arrives in the nonvolatile regime. The same procedure has been used in previous work with alloyed memristors^27^ and can be found in Supplementary Table 4.

Upon reaching a current limitation of 1 mA, the memristor turns nonvolatile and the training starts. 40 $\:I\left(V\right)$ cycles are shown (grey) together with the calculated median cycle (red), Fig. 4b. The nonvolatile characteristic of the device is observed by the gradual decrease in current during the negative polarity section of the $\:I\left(V\right)$ sweep. The gradual reduction of current indicates a slow reset of the device to the HRS. Therefore, the triangular voltage input sweep needed to be adapted to ensure a reset of the device. A higher negative polarity of − 2 V was chosen. However, the other measurement parameters such as sweep speed and direction are constant throughout all measurements. In the nonvolatile regime, the set voltage characteristics improved significantly with a lower mean of 0.35 V and a standard deviation of only 0.03 V. The higher current limitation leads to the formation of wider and long-term stable filament, improving both the set voltage and the cycle-to-cycle variability. After these training cycles in the nonvolatile regime, the device is then returned to the volatile operation regime by adapting the compliance current directly without going through intermediate steps.

The volatile operation of the device after nonvolatile training is shown in Fig. 4c. 40 $\:I\left(V\right)$ cycles are shown (grey) with the calculated median cycle (blue). As can be also seen by the V_set_ distribution, the switching characteristics drastically improved. The mean set voltage reduced to 0.23 V from the initial 0.77 V and the distribution narrowed from 0.22 V down to 0.01 V indicating a significant improvement of cycle-to-cycle variability. The proposed training was repeated with additional devices yielding similar results (Supplementary Fig. 8). For 40 $\:I\left(V\right)$ cycles on five devices each, the volatile operation improved. The set point was reduced from a mean V_set_ of 1.2 V to 0.2 V and the standard deviation improved from an initial 0.53 V to 0.03 V. A plausible explanation for the improved volatile switching is, that parts of the previously stable nonvolatile filament are remaining in the switching medium, thus creating a preferred filament growth path and reducing bot variability and set voltage. The reduction of the HRS resistance by about one order of magnitude compared to the initial volatile switching (Supplementary Fig. 7) supports this hypothesis. The measurement of a device in the volatile regime over 400 cycles did not yield comparable results (Supplementary Tables 2, 3).

To investigate the configurability between volatile and nonvolatile operation, we switched between the operation modes seven times measuring 40 $\:I\left(V\right)$ cycles each without intermediate current compliance steps after the initial training, Fig. 4d. The cycles are shown in grey with the calculated median highlighted in blue or red. In the volatile regime, the current is again limited to 10 nA and in the nonvolatile to 1 mA. These measurements indicate that it is possible to reliably operate the alloyed memristor in either of the two different retention regimes and additionally switch between them. To show the repeatability of the process, measurements of two additional devices performing the operation switching can be found in the supplementary information (Supplementary Fig. 9).

## Conclusions

In conclusion, we have demonstrated a multipurpose and reconfigurable alloyed AgSn memristor that can mimic both neuron and synaptic-like functionalities by operating in a volatile (neuronal) or nonvolatile (synaptic) regime. The operation regime is selected by control of the compliance current. Additionally, we demonstrate a training method, which significantly improves switching variability in the volatile regime.

In more detail, in the volatile operation regime, we experimentally demonstrated the neuron-like properties of the alloyed memristor. With a simple circuit consisting of a high resistance in series with the memristor, we implement neuron functions including “All-or-None”, “Integrate”, and “Integrate and Fire” by changing the input pulse signal. Thereby, we demonstrate the coexistence of multiple neuron-like functions without the typical need for more complex circuit elements such as capacitances. In the nonvolatile regime, we show the implementation of synaptic weights. This implementation consists again of a simple circuit with only a resistance in series with the memristor. Contrary to the neuron case, the series resistance is low, ensuring nonvolatile operation of the memristor necessary for long-term synaptic weights. Subsequent potentiation and depression measurements experimentally validate synaptic weight implementation with a good cycle-to-cycle repeatability on the same memristor. Finally, we demonstrate a training procedure by switching between the operation regimes. The mean switching voltage and its standard deviation are drastically reduced by a factor larger than 2 and 7 respectively in the volatile regime. We attribute these improvements to the development of a preferred formation path during the nonvolatile operation.

This work demonstrates the possibility to implement both a neuron and a synapse in a single device by operating in different current regimes. Additionally, we show that switching between these operations regimes is possible. We achieved both neuron and synaptic functionalities on the same device by only using series resistances to limit the device current. Future implementation in a device array in a 1T1R (one transistor one memristor) configuration presents a promising strategy, where the transistor can be employed to selectively control device operation. This property of the alloyed memristor makes it an excellent candidate for integration in beyond Moore architectures, such as neuromorphic computing, where multipurpose and reconfigurable hardware is desirable. 

## Sample fabrication

The material stack of the measured devices is shown in Supplementary Fig. 1. The substrate consists of a thermal SiO_2_ layer on top of Si. Initially, the bottom Pt electrode is patterned with electron beam lithography. Metals (3 nm Ti/47 nm Pt) are then deposited with electron beam evaporation. After lift-off, the structures are polished under a low incident angle with argon ion milling. The switching medium SiO_2_ is then deposited with atomic layer deposition at 300 °C with a thickness of 5 nm. The top metal alloy electrode is again patterned with electron beam lithography. The active metal (1 nm Ag/1 nm Sn/18 nm Ag) followed by an 80 nm capping layer of Pt are deposited with magnetron sputtering. In a final step, contact pads are patterned with a photomask. Reactive ion etching is used to access the bottom electrode through the switching medium. The final contact metal layers are deposited with electron beam evaporation (3 nm Ti/77 nm Au) followed by a lift-off process.

## Electrical measurement and data analysis

For the pulsed measurement, an arbitrary wave generator (HP AWG 33120 A), series resistance, transimpedance amplifier (FEMTO DHPCA-100), and an oscilloscope (Keysight MSO9104A) are used. The AWG is programmed with the desired input signal, which is then applied to the series resistance and the memristor. The current-limiting series resistance enables operation in the different retention regimes.

For the $\:I\left(V\right)$ characterization, a source measurement unit (SMU) is directly connected to the device. The SMU can directly limit the current through the device, which can be used to access the different operation regimes and additionally protect the device from destruction due to high currents. A triangular voltage signal is applied with different ranges depending on the operation regime (volatile: −0.5 V to 3 V, nonvolatile: −2 V to 2 V). The sweeping speed of all $\:I\left(V\right)$ measurements were kept at a constant 1 V/s. However, due to the auto range feature of the SMU there are slight variations in the sweeping speed. The device is trained by starting $\:I\left(V\right)$ sweeps at low compliance currents (10 nA) and steadily increasing the limit until the nonvolatile range (1 mA) is reached. Investigation into the SMU behavior showed that at the start and end of a sweep at 0 V the applied voltage is in fact a few µV which leads to a non-zero current especially in the LRS.

For the pulse data analysis, when no voltage is applied, the read-out current was set to 0 A to increase the readability of the figures. Additionally, a low-pass filter with a cut-off at (10 MHz) was used to increase the readability of the conductance subfigures.

For the DC data analysis, the V_set_ of a device is defined as the point where the device reaches 90% of the set compliance current.

## Atomic diffusion simulations

To investigate the role of the alloyed Sn material, five samples of amorphous SiO_2_ (each with 2940 atoms) were generated with a melt and quench approach using the PMMCS interatomic potential^41^ within the LAMMPS molecular dynamics package^42^. A cubic cell matching the experimental density of 2.2 g/cm^3^ was obtained by performing non-equilibrium molecular dynamics^43^ and deforming the cell during the melt phase at 4000 K. After quenching, a single charged metallic ion, either Ag or Sn, was inserted at a random site of the sample. Diffusion trajectories were extracted by following the trajectory of the metallic atom, including the periodic boundaries, at 1500 K for 20 ns.

## Supplementary Information

Below is the link to the electronic supplementary material.

Supplementary Material 1

## Data Availability

The data is available from the corresponding author Elias Passerini on reasonable request via email elias.passerini@ief.ee.ethz.ch.

## Code availability

The code is available from the corresponding author Elias Passerini on reasonable request via email elias.passerini@ief.ee.ethz.ch.
