# Supplementary material for: Reconfigurable artificial neuron and synapse enabled through a single alloyed memristor
Source: Sci Rep. 2025 Aug 13;15:29745. doi: 10.1038/s41598-025-15251-x (PMC12350929; doi:10.1038/s41598-025-15251-x)
Supplement: Supplementary file 1 — Supplementary Material 1 [file 41598_2025_15251_MOESM1_ESM.docx]

Reconfigurable Artificial Neuron and Synapse
 Enabled Through a Single Alloyed Memristor

Elias Passerini^(1)*^, Mila Lewerenz^(1)^, Arnaud Schneuwly^(1^,^)^ Nadia Jimenez Olalla^(1)^, Markus Fischer^(1)^, Raphael Gisler^(1)^, Luiz Felipe Aguinsky^(2,4)^, Alexandros Emboras^(2)^, Yuriy Fedoryshyn^(1)^, Mathieu Luisier^(2)^, Thomas Schimmel^(3)^, Miklós Csontos^(1)^, Ueli Koch^(1)^ & Juerg Leuthold^(1)*^

*(1) Institute of Electromagnetic Fields (IEF), ETH Zurich, 8092 Zurich, Switzerland*

*(2) Integrated Systems Laboratory (IIS), ETH Zurich, 8092 Zurich, Switzerland*

*(3) Institute of Applied Physics (APH), Karlsruhe Institute of Technology, 76131 Karlsruhe, Germany*

*(4) DeepNano Group, James Watt School of Engineering, University of Glasgow, G12 8LT Glasgow, UK*

* Email Correspondence: [elias.passerini@ief.ee.ethz.ch](mailto:elias.passerini@ief.ee.ethz.ch), juerg.leuthold@ief.ee.ethz.ch

**SUPPLEMENTARY DATA AND FIGURES**

# Device Structure and Capacitance

**Supplementary Figure 1** SEM image and material layer stack of the alloyed memristor. (**a**) SEM image of the AgSn-alloyed memristor with a bottom electrode consisting of Pt and a top electrode consisting of AgSn and Pt. The scalebar is 10 μm. (**b**) The substrate consists of thermal SiO_2_ on top of Si. The bottom electrode consists of Pt and the switching medium is atomic layer deposited SiO_2_. The alloyed top electrode is a mix of Ag and Sn with a total layer thickness of 20 nm. The 80 nm Pt capping layer protects the top electrode. (**c**) cross section image of the alloyed memristor. The top electrode AgSn/Pt and the bottom electrode Pt sandwich the switching medium SiO_2_. The scale bar is 500 nm.


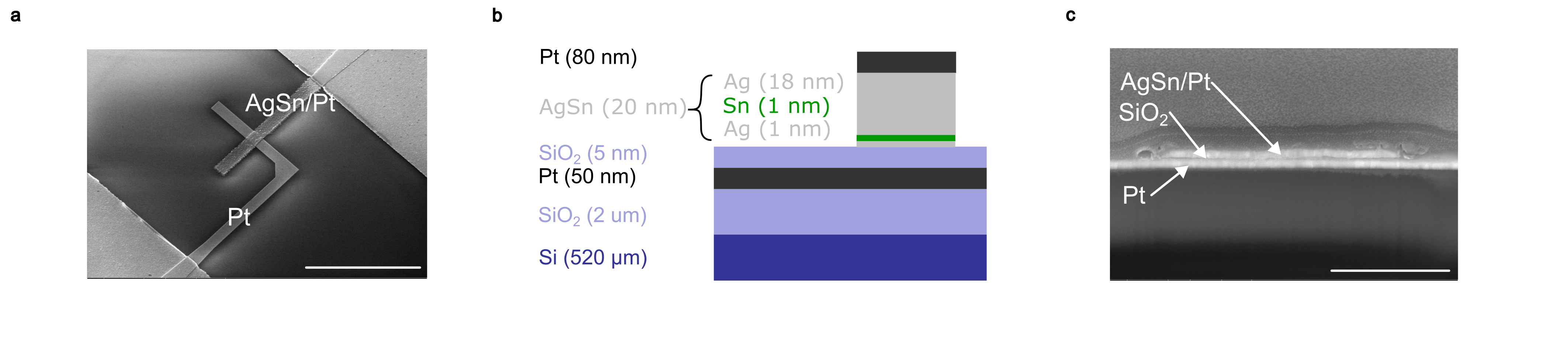


With the device area $A$ of the AgSn-alloyed memristor (1 μm x 1 μm), the electrode distance $d$ (5 nm), and the relative dielectric constant of SiO_2_ (3.9) one can estimate the device capacitance:

$$C=\epsilon\frac{A}{d}\approx3.9*8.85*{10}^{-12}Fm^{-1}\frac{\left( 1*{10}^{-6}m \right)^{2}}{5*{10}^{-9}m}\approx6.9*{10}^{-15}F$$

# AgSn Diffusion


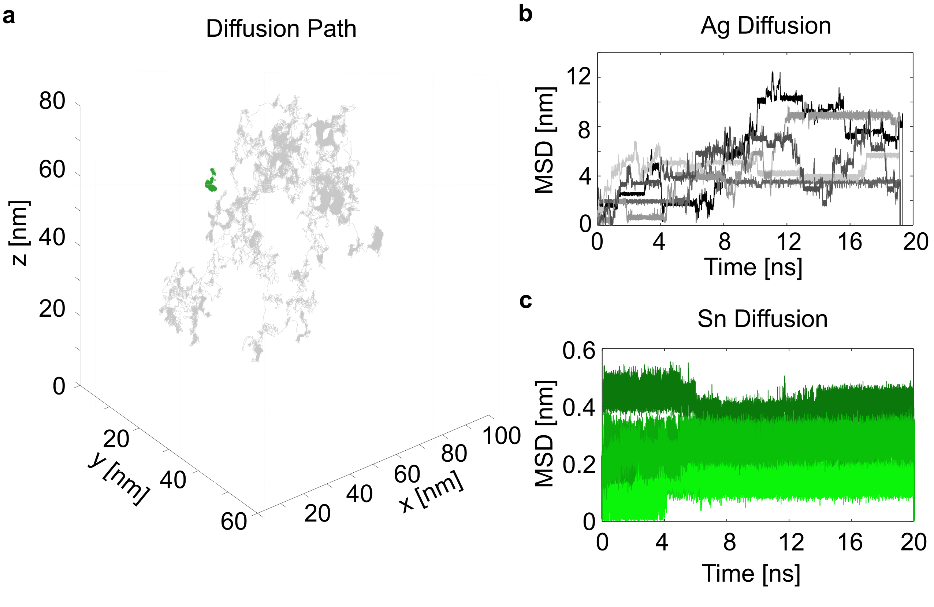


**Supplementary Figure 2** Diffusion of Ag and Sn in amorphous SiO_2_ at 1500 K. (**a**) Example diffusion path of Ag (grey) and Sn (green) at 1500 K. (**b**) Mean squared displacement from the starting point of five Ag ions at 1500 K. (**c**) Mean squared displacement from the starting point of five Sn ions at 1500 K showing that the Sn diffuses less compared to the Ag.

# Retention


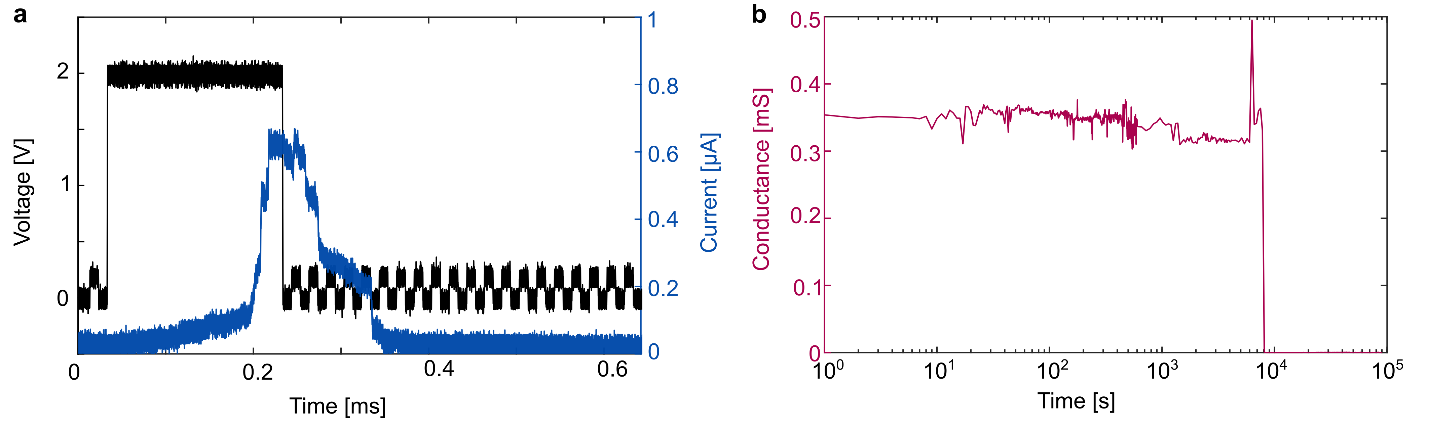


**Supplementary Figure 3** Retention measurement for volatile and nonvolatile operation. (**a**) To operate the device in a volatile operation mode the current is limited by a 2 MΩ series resistance. After the set pulse (2 V, 200 μs) the device turns on. However, after 80 μs the device current drastically reduces indicating a reset of the device. (**b**) To operate the device in the volatile regime a series resistance of 1 kΩ was chosen. After an initial set the device conductance is read by a read pulse (0.1 V, 10 μs). The conductance state does not significantly change until 1000 s. After 6000 s the conductance changes drastically and the device resets.

# Set Time Measurements


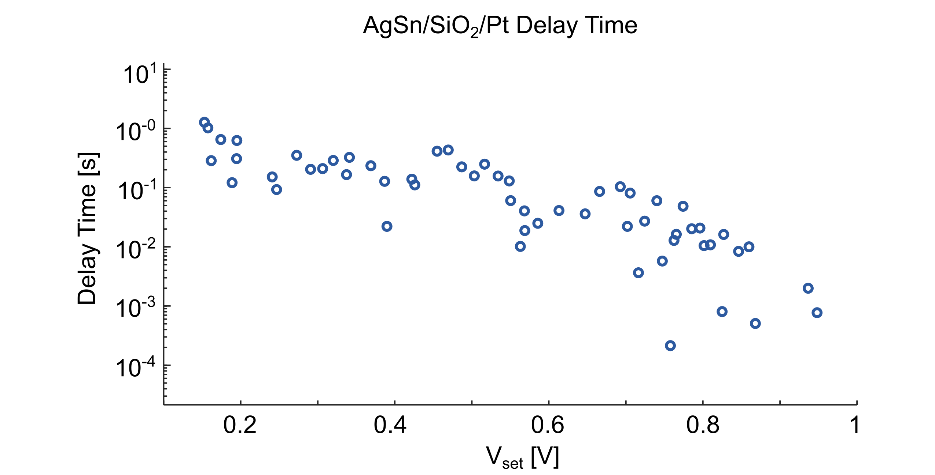


**Supplementary Figure 4** .Switching delay time in relation to the set pulse amplitude. The AgSn/SiO2/Pt devices exhibit an exponential relation between voltage amplitude and delay time where the device changes from the HRS to the LRS.

# Neuron Operation


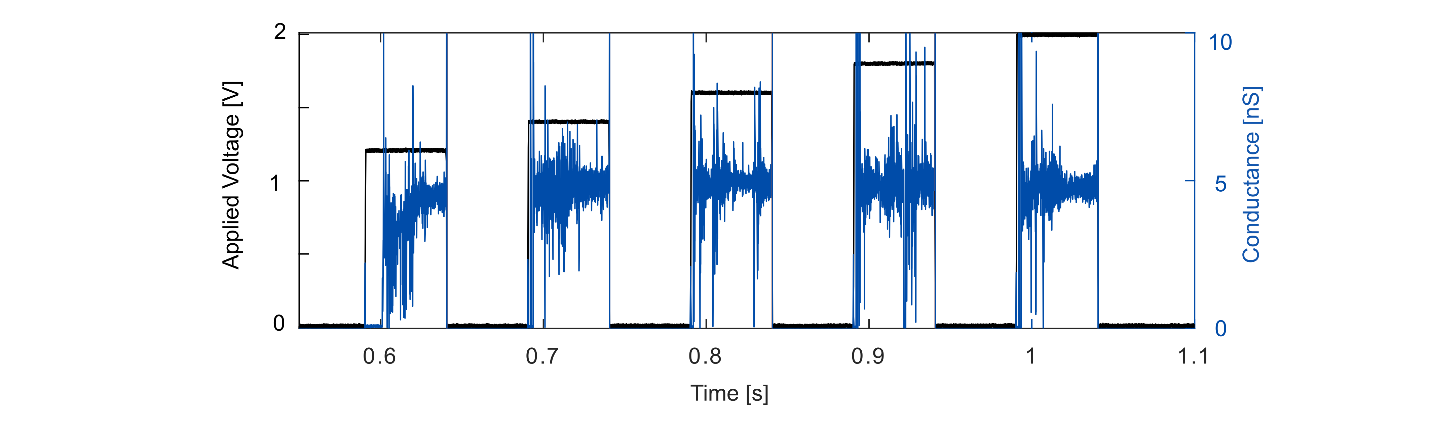


**Supplementary Figure 5** All-or-none conductance level for pulses above the threshold in linear scale


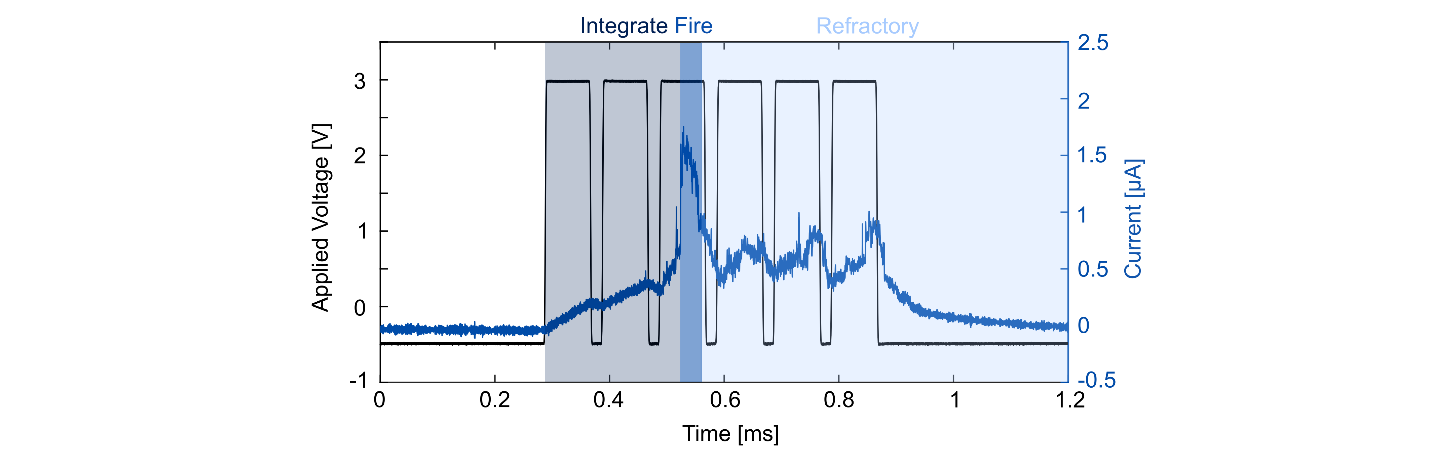


**Supplementary Figure 6** IF measurement with a pulse amplitude of 3 V and length of 100 μs and a duty cycle of 80%. After “Integration” the device “fires” which is followed by the “Refractory” period. During the integration step “leakage” is visible between the pulses.


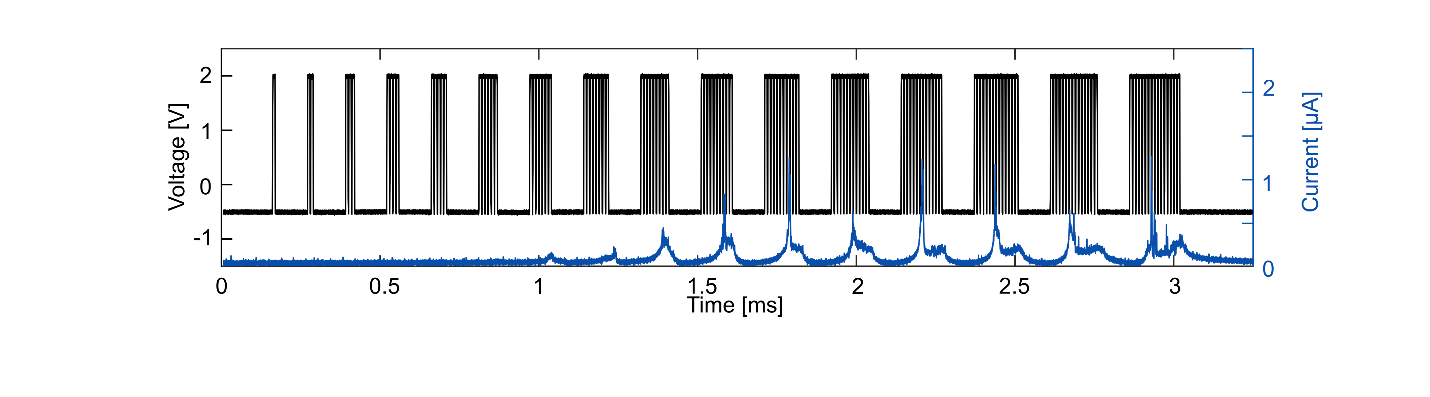


**Supplementary Figure 7** IF measurement with 2.5 V amplitude, -0.5 V bias offset, 10 μs pulse length and a duty cycle of 80%. This measurement indicates that the spiking frequency could be controlled by the negative offset as there is only one spiking event per pulse train compared to the two in Fig. 2 (i), which has a -1 V bias offset.

# Changing Operation Mode

| **Cycle Number:** | **1-10** | **11-20** | **21-30** | **31-40** |
| --- | --- | --- | --- | --- |
| Mean Set Voltage $\left[ V \right]$: | 0.61 | 0.75 | 0.80 | 0.89 |
| **Cycle Number:** | **1-10** | **11-20** | **21-30** | **31-40** |
| Standard Deviation Voltage $\left[ V \right]$: | 0.2 V | 0.25 V | 0.12 V | 0.17 V |

**Supplementary Table 1** Mean and standard deviation for the first 40 cycles of a device showing no improved device performance.

| **Cycle Number:** | **1-20** | **21-40** | **41-60** | **61-80** | **81-100** | **101-120** | **121-140** | **141-160** | **161-180** | **181-200** |
| --- | --- | --- | --- | --- | --- | --- | --- | --- | --- | --- |
| Mean Set Voltage $\left[ V \right]$: | 2.53 | 2.41 | 2.52 | 2.58 | 2.71 | 1.52 | 1.08 | 1.32 | 1.35 | 1.46 |
| **Cycle Number:** | **201-220** | **221-240** | **241-260** | **261-280** | **281-300** | **301-320** | **321-340** | **341-360** | **361-380** | **381-400** |
| Mean Set Voltage $\left[ V \right]$: | 1.69 | 2.51 | 2.45 | 2.16 | 2.48 | 2.10 | 1.83 | 2.09 | 2.32 | 2.13 |

**Supplementary Table 2** Mean set voltage of an AgSn memristor over 400 cycles shown for 20 subsequent cycles each. Showing no clear improvement in the device performance.

| **Cycle Number:** | **1-20** | **21-40** | **41-60** | **61-80** | **81-100** | **101-120** | **121-140** | **141-160** | **161-180** | **181-200** |
| --- | --- | --- | --- | --- | --- | --- | --- | --- | --- | --- |
| Standard Deviation $\left[ V \right]$: | 0.23 V | 0.20 V | 0.13 V | 0.18 V | 0.19 V | 0.55 V | 0.17 V | 0.13 V | 0.20 V | 0.24 V |
| **Cycle Number:** | **201-220** | **221-240** | **241-260** | **261-280** | **281-300** | **301-320** | **321-340** | **341-360** | **361-380** | **381-400** |
| Standard Deviation $\left[ V \right]$: | 0.37 V | 0.29 V | 0.36 V | 0.29 V | 0.25 V | 0.55 V | 0.14 V | 0.23 V | 0.37 V | 0.38 V |

**Supplementary Table 3** Standard deviation of the set voltage of an AgSn memristor over 400 cycles shown for 20 subsequent cycles each. Showing no clear improvement in the device performance.

| **Compliance Current** | **Cycles** | **Ramp** | **Voltage Range** |
| --- | --- | --- | --- |
| 10 nA | 40 | 1 V/s | -0.5 V - 3 V |
| 100 nA | 20 | 1 V/s | -0.5 V - 3 V |
| 1 μA | 40 | 1 V/s | -0.5 V - 3 V |
| 10 μA | 20 | 1 V/s | -0.5 V - 3 V |
| 100 μA | 20 | 1 V/s | -0.5 V - 3 V |
| 1 mA | 40 | 1 V/s | -2 V - 2 V |

**Supplementary Table 4** The training procedure of the devices with increasing compliance currents from the volatile to the non-volatile regime.


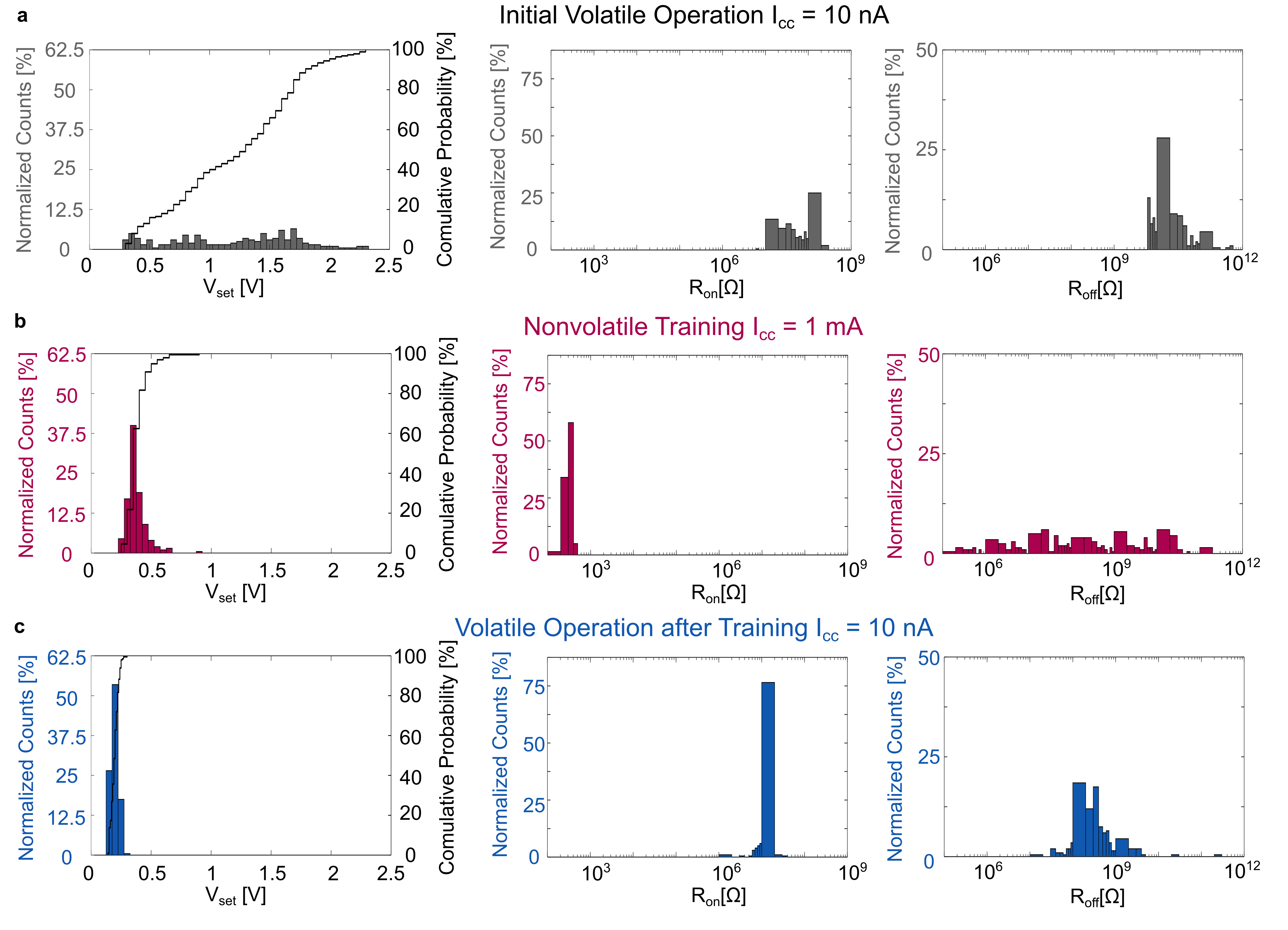


**Supplementary Figure 8** Improved volatile operation through extended training. (**a**) Initial volatile operation at a compliance current of 10 nA. Set voltage V_set_ five devices and 40 $I(V)$cycles for each device shown together. The set voltage spread is significantly large. On/off resistance R_on_/R_off_ extracted from the same measurement. (**b**) Nonvolatile training of the same devices at a compliance current of 1 mA. The set voltage mean and spread reduced significantly. The on resistance is below kΩ indicating a long-term stable filament. The off resistance spread increased compared to the volatile training. (**c**) Volatile operation of the same devices at a compliance current of 10 nA after nonvolatile cycling. Compared to the initial measurements, the set voltage mean (μ(V_set_): 1.2 V→0.2 V) and spread (σ(V_set_): 0.52 V→0.03 V) significantly improved, thus reducing both device-to-device and cycle-to-cycle variability. The off resistance reduced by roughly one order of magnitude indicating the remainder of a conducting path.


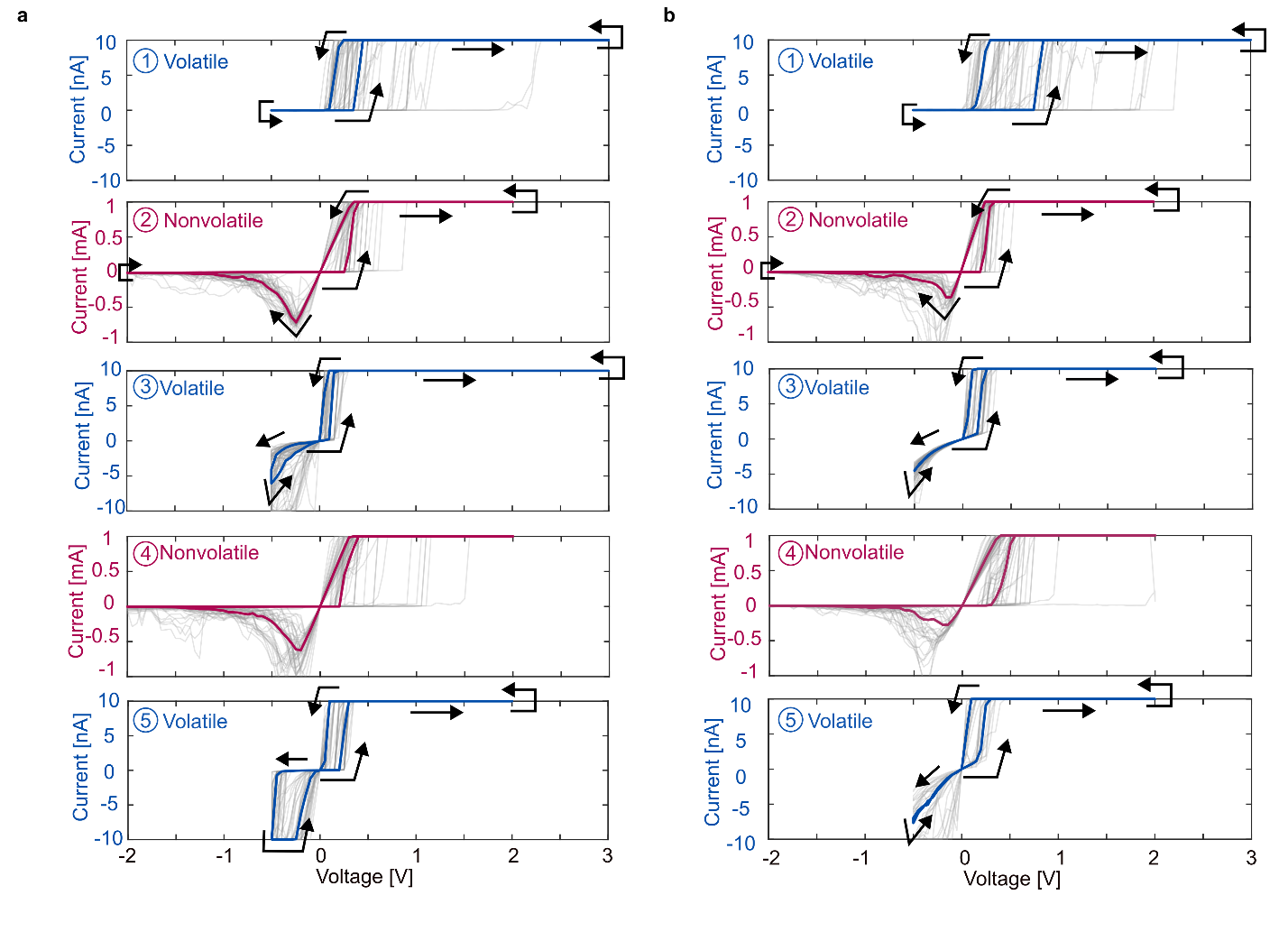


**Supplementary Figure 9** Two additional devices showing the ability to switch between operation modes. Sweep directions are indicated by the arrows. The devices were initially measured in the volatile operation regime at a low compliance current (10 nA) by applying 40 I(V) cycles (grey) with their mean cycle highlighted (blue). This is followed by an operation change to the nonvolatile regime, where again 40 I(V) cycles were measured with their mean cycle highlighted (red). The switching between operation modes was executed four times for each of the devices. (**a**) Device 1. This device switching characteristic turned nonpolar with subsequent operation mode changes eg. The devices started to switch with negative polarity. (**b**) Device 2.
